# Utility of Endobronchial Ultrasound-Guided Transbronchial Needle Aspiration in Diagnosis of Intrathoracic Lymphadenopathy in Patients with Human Immunodeficiency Virus Infection

**DOI:** 10.1155/2015/257932

**Published:** 2015-01-14

**Authors:** Audrey Yan Yi Han, Aik Hau Tan, Mariko Siyue Koh

**Affiliations:** Department of Respiratory and Critical Care Medicine, Singapore General Hospital, 20 College Road, Academia, Singapore 169856

## Abstract

*Objective*. Intrathoracic lymphadenopathy (LAD) in patients with Human Immunodeficiency Virus (HIV) infection is common, with wide-ranging diagnoses, from benign to malignant causes. Endobronchial Ultrasound-guided Transbronchial Needle Aspiration (EBUS-TBNA) is a relatively new technology with established applications in lung cancer, sarcoidosis, and tuberculosis. We sought to find out whether the addition of EBUS-TBNA to the diagnostic algorithm for LAD in HIV patients will reduce the need for mediastinoscopy. *Methods*. Retrospective chart review of all EBUS-TBNA procedures performed in our centre from August 2008 to December 2012. *Results*. 513 patients had EBUS-TBNA performed during this period. We identified nine HIV-infected patients who had LAD of unknown cause and underwent EBUS-TBNA. The procedure reduced the need for mediastinoscopy in eight patients (89%). *Conclusions*. Potential mediastinoscopies can be avoided by utilising EBUS-TBNA in HIV patients with LAD.

## 1. Introduction

Intrathoracic lymphadenopathy (LAD) is a challenging problem in patients infected with Human Immunodeficiency Virus (HIV). Jasmer et al. demonstrated an incidence of 35% based on routine computed tomography (CT) scan, performed for a cohort of 318 patients with HIV infection [1]. Diagnoses are extensive, ranging from commoner diagnoses of Mycobacterial infections (tuberculous and nontuberculous) and malignancies (lymphoma, primary lung cancer, Kaposi's sarcoma) to less common diagnoses of reactive lymphadenopathy in infection (bacterial, fungal,* Pneumocystis jirovecii* pneumonia previously known as* Pneumocystis carinii* pneumonia) and nonspecific lymphoid hyperplasia [1, 2].

Conventionally, patients would undergo mediastinoscopy or open biopsy via thoracotomy, which incur higher morbidity and a risk of general anaesthesia as compared to bronchoscopic procedures [3]. Conventional Transbronchial Needle Aspiration (TBNA) via bronchoscopy was an alternative method of diagnosis, but with a diagnostic rate of only 52% when performed in this group of patients [4]. Endobronchial Ultrasound-guided Transbronchial Needle Aspiration (EBUS-TBNA) is a relatively new technology which uses real-time linear ultrasound imaging to guide intrathoracic lymph node and lung mass tissue sampling during bronchoscopy. Its utility in the diagnosis of lung cancer [5], sarcoidosis [6], and tuberculosis [7] has been proven; in particular, it has been shown to have significantly better sensitivity and yield than conventional TBNA for lung cancer and sarcoidosis [5, 6]. Our aim was to find out if addition of EBUS-TBNA into the diagnostic algorithm for LAD in HIV patients will reduce the need for mediastinoscopy.

## 2. Methods

Retrospective chart review of all EBUS-TBNA procedures performed in our centre from August 2008 to December 2012 was carried out. Completeness of data was ensured with the use of a centralized database that was set up at the time of introduction of EBUS-TBNA to our institution. The study was approved by our Centralized Institutional Review Board (2008/458/B). All cases of HIV infection were confirmed by Western blot. Demographic data, clinical presentations, results, clinical management, and outcomes are summarised in Table 1.

Informed consent was obtained from each patient prior to the procedure. EBUS-TBNA was carried out in our endoscopy suite under local anaesthesia and moderate sedation with midazolam and/or fentanyl.

## 3. Results

513 patients had EBUS-TBNA performed during this period. We identified nine HIV-infected patients who had LAD of unknown cause that underwent EBUS-TBNA. The average age of these HIV-infected patients was 52 years (range: 40 to 73 years) and the average CD4 count was 197 (range: 2 to 433 cells/mm^3^). The most common presenting symptom was fever and the paratracheal lymph nodes were the most frequently sampled. The addition of EBUS-TBNA reduced the need for mediastinoscopy in eight out of nine patients (Table 1).

All 5 cases of Mycobacterial infection were treated with Mycobacterial therapy, with clinical and radiological improvement. One patient with small cell lung cancer died before treatment was commenced (Patient 1). One patient with diffuse large B-cell lymphoma received chemotherapy but died 10 weeks later from progressive disease (Patient 8) (Table 1).

Although an indeterminate result was recorded for Patient 2, he remains clinically well 22 months after EBUS-TBNA. Follow-up CT thorax 22 months later revealed stable mediastinal lymphadenopathy, partial resolution of axillary lymphadenopathy, and no new findings. Thus, he was deemed to have nonspecific mediastinal lymphadenopathy. Similarly, Patient 6 remains clinically well on follow-up 24 months after EBUS-TBNA.

One complication was encountered. Patient 1 had an uneventful bronchoscopic procedure, but desaturated and had an asystolic collapse 18 hours thereafter. Chest radiograph did not reveal any pneumothorax or new consolidation. He did not have any haemoptysis to suggest bleeding as a complication of the procedure. The cause of death was attributed to an acute myocardial event, as he had significant cardiovascular risk factors (diabetes, hypertension, smoker). The case was referred to the coroner's office but an autopsy was not performed.

## 4. Discussion

We found that EBUS-TBNA decreased the need for mediastinoscopy in eight out of nine (89%) HIV patients with LAD. EBUS-TBNA was able to diagnose four out of five cases of lymphadenopathy secondary to Mycobacterial infections, one case of diffuse large B-cell lymphoma and one case of small cell lung cancer. It was nondiagnostic in one case of tuberculosis (diagnosed by mediastinoscopy). Although a definitive diagnosis was not obtained in two patients, these patients had relatively small lymph node enlargement (≤15 mm), which likely represented an inflammatory reaction to HIV infection, rather than actual lymph node involvement (i.e., “true negative” EBUS-TBNA procedures). Stability in their conditions after 22 months and 24 months of follow-up further supports the conclusion that the lymphadenopathy observed was unlikely to be clinically significant.

To our knowledge, this is the first series describing the utility of EBUS-TBNA in the diagnosis of LAD in HIV-infected patients, and our findings support the utilisation of this bronchoscopic technique in this group of patients.

Although there was a patient who died 18 hours after bronchoscopy, this was likely due to the sedation and/or hypoxemia related to bronchoscopy, rather than EBUS-TBNA itself. Major complications from bronchoscopy are uncommon (0.08% to 0.5%), and so is mortality (up to 0.04%) [8]. Based on a nation-wide survey involving 520 centres in Japan, complications arising from EBUS-TBNA are also uncommon (1.2%), and mortality is rare (0.01%) [9]. The main complications were bleeding (0.68%) and infection (0.19%) [9]. Our patient's significant cardiovascular risk factors of diabetes, hypertension, and smoking, as well as the events leading to his demise, strongly suggest an acute myocardial event. In general, complication and mortality rates from bronchoscopy (0.08–0.5% and 0.04%, resp.) and EBUS-TBNA (1.2% and 0.01%, resp.) are still comparable to or lower than those of mediastinoscopy (1.07% and 0.05%, resp.) [3]. In addition, EBUS-TBNA does not require general anaesthesia or an inpatient hospitalization in most centres.

The main limitations of our study are its retrospective nature and small numbers. However, completeness of data is ensured by the centralized database that was set up at the time of introduction of EBUS-TBNA to our institution, as well as cross-checking of results with the Electronic Health Records. Secondly, patients referred for EBUS-TBNA were screened for HIV infection only when there was clinical suspicion of the disease. We recognise that there may be patients who have undergone EBUS-TBNA with concomitant undiagnosed HIV infection. Nevertheless, as a tertiary referral centre, we have a low threshold for ordering HIV testing in patients. In fact, 33.3% (171 of 513 patients) of our EBUS-TBNA cohort had undergone HIV testing. Therefore, we believe that we are unlikely to have underdiagnosed HIV infection in our cohort of patients, and that our findings are representative of real-life clinical practice.

In conclusion, it appears that potential mediastinoscopies can be avoided by utilising EBUS-TBNA in HIV patients with intrathoracic lymphadenopathy.

## Figures and Tables

**Table 1:** Demographic, clinical, and radiographic findings.

Number	Age/sex	Smoker	Time elapsed since HIV diagnosis/current treatment	Presenting symptoms/duration	Findings on computed tomography of thorax/other significant imaging results	CD4 (cells/mm^3^)	Lymph node sampled^a^ (size, mm)	Histology/cytology	Microbiological results	Treatment/outcome	Need for mediastinoscopy (final diagnosis)
1	73/M	Yes	5 years/ART	Fever, cough, purulent sputum/1 week	Right hilar mass, mediastinal lymphadenopathy, bilateral pulmonary nodules (<10 mm)/multiple hepatic nodules, bilateral adrenal nodularity, lytic bone lesions	386	4R (30)	Small cell lung cancer	TB culture from TBNA negative	Not treated/demised	No (small cell lung cancer)

2	59/M	Yes	3 months/untreated	Hemoptysis/2 weeks	Mediastinal and bilateral axillary lymphadenopathy, fibrobullous disease in both upper lobes	433	4R (6) 4L (12)	Blood	TB culture from TBNA negative BAL cultures negative	Surveillance	No (nonspecific)

3	39/F	No	13 years/defaulted treatment 3 years	Fever, weight loss/1 week	Mediastinal and right hilar lymphadenopathy, 2 nonspecific pulmonary nodules (<10 mm)	2	4R (20)	Lymphocytes	TB culture from TBNA: MAC Bacterial and fungal cultures from TBNA negative	Treated for disseminated MAC/resolved	No (disseminated MAC)

4	41/M	Yes	New diagnosis/untreated	Fever, cough, purulent sputum, weight loss/2 weeks	Necrotic left hilar mass, mediastinal lymphadenopathy, left lower lobe nodules	127	7 (30)	Lymphocytes (mediastinoscopy: necrotising granulomatous inflammation)	TB culture from TBNA negative BAL cultures: MTC Mediastinoscopy cultures: MTC	Treated for MTC/resolved	Yes (TB)

5	40/M	No	8 months/ART	Cough, nonpurulent sputum/1 month	Necrotic mediastinal and right hilar lymphadenopathy, patchy consolidation and tree-in-bud nodules in right lung/necrotic retroperitoneal lymphadenopathy	102	4R (30)	Necrotic tissue Acid fast bacilli present	TB culture from TBNA negative BAL cultures negative Cervical lymph node culture: MAC	Treated for disseminated MAC/resolved	No (disseminated MAC)

6	47/M	Yes	4 years/ART	Anterior chest wall pain and swelling/1 week	Enlarged precarinal node, no lesion seen in the anterior chest wall	11	4L (15)	Necrotic tissue	TB culture from TBNA negative	Surveillance	No (nonspecific)

7	50/M	Yes	New diagnosis/untreated	Fever, appetite, and weight loss/1 month	Necrotic mediastinal and right hilar lymphadenopathy/necrotic intra-abdominal lymphadenopathy	83	2R (11)	Necrotic tissue	TB culture from TBNA: MTC Fungal culture from TBNA negative	Treated for MTC/resolved	No (TB)

8	68/M	Yes	New diagnosis/untreated	Cough, appetite, and weight loss/2 weeks	Mediastinal and bilateral hilar lymphadenopathy, left upper lobe mass/ intra-abdominal lymphadenopathy	346	4L (12) 7 (21) 10L (7)	Diffuse large B-cell lymphoma	TB culture from TBNA negative BAL cultures negative	Chemotherapy/demised	No (diffuse large B-cell lymphoma)

9	49/M	Yes	New diagnosis/untreated	Cough, nonpurulent sputum/3 months Fever, appetite, and weight loss/3 weeks	Necrotic mediastinal, right hilar and right supraclavicular lymphadenopathy, bilateral pulmonary nodules (<10 mm)	280	4R (30)	Blood	TB culture from TBNA: MTC	Treated for MTC/resolved	No (TB)

HIV: Human Immunodeficiency Virus, ART: antiretroviral therapy, TBNA: Transbronchial Needle Aspiration, BAL: bronchoalveolar lavage, MAC: mycobacterium avium complex, TB: tuberculosis, MTC: mycobacterium tuberculosis complex.

^a^According to the International Association for the Study of Lung Cancer lymph node map (2009). 2R: right upper paratracheal, 4R: right lower paratracheal, 4L: left lower paratracheal, 7: subcarinal, 10L: left hilar.
